# Antibody levels to recombinant VAR2CSA domains vary with *Plasmodium falciparum* parasitaemia, gestational age, and gravidity, but do not predict pregnancy outcomes

**DOI:** 10.1186/s12936-018-2258-9

**Published:** 2018-03-09

**Authors:** Michal Fried, Jonathan D. Kurtis, Bruce Swihart, Robert Morrison, Sunthorn Pond-Tor, Amadou Barry, Youssoufa Sidibe, Sekouba Keita, Almahamoudou Mahamar, Naissem Andemel, Oumar Attaher, Adama B. Dembele, Kadidia B. Cisse, Bacary S. Diarra, Moussa B. Kanoute, David L. Narum, Alassane Dicko, Patrick E. Duffy

**Affiliations:** 1 Laboratory of Malaria Immunology and Vaccinology, National Institute of Allergy and Infectious Diseases, NIH, Rockville, MD USA; 2 Center for International Health Research, Rhode Island Hospital, Brown University Medical School, Providence, RI USA; 3 Biostatistics Research Branch, National Institute of Allergy and Infectious Diseases, NIH, Rockville, MD USA; 4 Malaria Research & Training Center, Faculty of Medicine, Pharmacy and Dentistry, University of Sciences Techniques and Technologies of Bamako, P.O Box 1805, Bamako, Mali

**Keywords:** Placental malaria, VAR2CSA, Birth weight, Pregnancy loss, Preterm delivery, Anaemia

## Abstract

**Background:**

Maternal malaria is a tropical scourge associated with poor pregnancy outcomes. Women become resistant to *Plasmodium falciparum* pregnancy malaria as they acquire antibodies to the variant surface antigen VAR2CSA, a leading vaccine candidate. Because malaria infection may increase VAR2CSA antibody levels and thereby confound analyses of immune protection, gravidity-dependent changes in antibody levels during and after infection, and the effect of VAR2CSA antibodies on pregnancy outcomes were evaluated.

**Methods:**

Pregnant women enrolled in a longitudinal cohort study of mother-infant pairs in Ouelessebougou, Mali provided plasma samples at enrollment, gestational week 30–32, and delivery. Antibody levels to VAR2CSA domains were measured using a multiplex bead-based assay.

**Results:**

Antibody levels to VAR2CSA were higher in multigravidae than primigravidae. Malaria infection was associated with increased antibody levels to VAR2CSA domains. In primigravidae but not in secundigravidae or multigravidae, antibodies levels sharply declined after an infection. A relationship between any VAR2CSA antibody specificity and protection from adverse pregnancy outcomes was not detected.

**Conclusions:**

During malaria infection, primigravidae acquire short-lived antibodies. The lack of an association between VAR2CSA domain antibody reactivity and improved pregnancy outcomes suggests that the recombinant proteins may not present native epitopes targeted by protective antibodies.

**Electronic supplementary material:**

The online version of this article (10.1186/s12936-018-2258-9) contains supplementary material, which is available to authorized users.

## Background

Malaria during pregnancy is a major public health problem, associated with severe maternal anaemia, low birthweight (LBW) delivery, preterm delivery (PTD), pregnancy loss and infant mortality [1]. During pregnancy, *Plasmodium falciparum* parasites sequester in the placenta and adhere to chondroitin sulfate A (CSA) expressed on the surface of syncytiotrophoblast [2]. Infected erythrocyte (IE) adhesion to CSA is mediated by VAR2CSA, a member of the *P. falciparum* erythrocyte membrane protein family (PfEMP1) that is preferentially expressed by placental parasites and parasites selected to bind CSA [3–5].

In areas of stable malaria transmission, primigravidae are at highest risk of *P. falciparum* pregnancy malaria and women develop resistance over 1–2 pregnancies that controls infection and prevents severe sequelae [6, 7]. This unique gravidity-dependent epidemiology correlates with the acquisition of immunity to placental parasites, and efforts to develop a placental malaria vaccine (PMV) that mimics naturally acquired immunity are on-going (reviewed in [8]). VAR2CSA is currently the leading candidate for a PMV. VAR2CSA is a large protein of about 350kD composed of 6 Duffy binding-like (DBL) domains together with inter-domain regions. Its large size and sequence variation pose unique challenges in designing a vaccine based on VAR2CSA [8].

Despite a central role in placental malaria pathogenesis, the evidence that VAR2CSA is targeted by protective antibodies has been inconsistent. Several studies compared primigravid and multigravid women for their seroreactivity to various VAR2CSA domains. Most studies reported that multigravidae have higher antibody levels to the relatively immunogenic DBL5 domain, while parity-dependent reactivity to other domains was less consistent [8]. A common finding across studies is that multigravidae have higher antibody levels to at least one VAR2CSA domain or to the full-length protein [3, 9–13]. Different results from studies of recombinant VAR2CSA seroreactivity patterns could be due to differences in allelic forms, domain boundaries, infection status, gestational age at the time of antibody measurement, or malaria prevalence in the study area.

Two studies in Cameroon reported that the breadth of reactivity to multiple domains, and levels of high avidity antibodies to full length recombinant VAR2CSA, were associated with a reduction in PM at delivery [14–16]. In Benin, high antibody levels at enrollment variously associated with improved outcomes: antibodies to full length recombinant VAR2CSA and to DBL3 were associated with a reduction in the number of infections during the follow up period; antibody to DBL3 with a reduction in placental malaria; and antibody to DBL1-DBL2 with a reduction in low birthweight [12]. Among Mozambican women who were infected at least once during pregnancy, above-the-median antibody levels to DBL3 and DBL6, as well as to malaria proteins MSP and AMA1 but not to DBL2, were associated with increased birthweight and gestational age [11].

Malaria infections boost antibody levels to the IE surface [17], and this could confound efforts to relate specific antibody titers to protection. In the current study of a longitudinal cohort of pregnant women, gravidity-dependent changes in antibody levels during and after malaria infection were evaluated. Further, relationships between VAR2CSA antibodies and preterm delivery or early pregnancy loss have not been studied. The present study examined whether VAR2CSA antibody levels predict pregnancy loss or preterm delivery risks, newborn gestational age and birthweight, and maternal anaemia risk.

## Methods

### Human subjects and clinical procedures

Pregnant women enrolled between November 2010 and October 2013 into a longitudinal cohort study of mother-infant pairs conducted in Ouelessebougou, Mali. The study site is located 80 km south of Bamako, an area of intense seasonal malaria transmission during the rainy season from July to December. Pregnant women aged 15–45 years without clinical evidence of chronic or debilitating illness were asked to participate in the study and gave signed informed consent after receiving a study explanation form and oral explanation from a study clinician in their native language. The protocol and study procedures were approved by the institutional review board of the National Institute of Allergy and Infectious Diseases at the US National Institutes of Health (ClinicalTrials.gov ID NCT01168271), and the Ethics Committee of the Faculty of Medicine, Pharmacy and Dentistry at the University of Bamako, Mali.

After enrollment, women underwent a clinical evaluation including a thorough medical history review of the current and previous pregnancies. The follow-up included scheduled monthly clinical examinations until 1 month post-delivery and at any time women felt sick. Blood smear samples for malaria infection and venous blood samples for the evaluation of the antibodies to VAR2CSA were collected at enrolment, week 30–32 and at delivery. Blood smear samples for malaria infection were also taken at scheduled and sick visits. Malaria infections were treated with quinine or with artemether–lumefantrine as clinically indicated. Women received 0–3 doses of intermittent preventive treatment with sulfadoxine–pyrimethamine (IPTp-SP), depending on recruitment trimester, number of doses received prior to enrollment, and on incidental parasitaemia which mandated treatment with other anti-malarial drugs. Gestational age was determined by ultrasound examination with Siui CTS-7700+ ultrasound scanner. Malaria infection was determined by thick blood smear. Infection was defined as the presence of any *P. falciparum* parasite in a peripheral or placental blood smear. At least 100 high power fields in the thick smear were examined before concluding that the result was negative. Submicroscopic infections were defined by nested PCR as previously described [18]. Miscarriage was defined as pregnancy ending at gestational week ≤ 28 weeks, stillbirth as a delivery of a non-viable baby at a gestational age of > 28 weeks, and early neonatal death as death occurring within 7 days of birth. Pregnancy loss included cases of miscarriage, stillbirth and early neonatal death. Preterm delivery was defined as birth prior to gestational age of 37 weeks. LBW was defined as a birth weight of < 2500 g. Small for gestational age (SGA) was defined as a birth weight below the tenth percentile for the gestational age and gender, based on a weight chart developed in Tanzania [19].

### Multiplex antibody assays

Recombinant VAR2CSA DBL domains were prepared based on alleles from laboratory parasite lines FCR3 and 3D7, and from the dominant VAR2CSA alleles identified by whole genome sequencing of maternal isolates M1010, M711, and M466 (Table 1). Eleven VAR2CSA fragments were cloned and expressed in *Escherichia coli* or baculovirus as previously described [20–22]. Recombinant glutamic acid rich protein (GARP, PlasmoDB ID PF3D7_0113000), a protein that is not unique to placental parasites, was included as a control. Antibody levels in plasma samples diluted 1:80 were measured using a multiplex bead-based assay (Bioplex: Bio-Rad, Irvine, CA), as previously described [23]. Antibody levels were expressed as median fluorescence intensity (MFI) after subtracting background reactivity with beads coated with BSA. Seropositivity was defined for each of the domains as the level above the mean + 2 SD obtained from 22 control plasma samples collected in the US.

**Table 1:** Recombinant DBL domains

Domain	Allele	Domain boundaries amino acids	Expression system
DBL2	FCR3	543–858	*E. coli*
ID1-ID2a	Maternal isolate 1010 (M1010)	428–1024^a^	Baculovirus
DBL3	FCR3	1220–1541	*E. coli*
DBL3-4	FCR3	1445–1989	*E. coli*
DBL4	FCR3	1583–1989	*E. coli*
DBL4	3D7	1570–1926	*E. coli*
DBL4	Maternal isolate 1010 (M1010)	1583–1989^a^	*E. coli*
DBL4	Maternal isolate 711 (M711)	1583–1989^a^	*E. coli*
DBL5	FCR3	2003–2281	*E. coli*
DBL5	Maternal isolate 1010 (M1010)	2003–2281^a^	*E. coli*
DBL5	Maternal isolate 466 (M466)	2003–2281^a^	*E. coli*

^a^Domain boundaries refers to the amino acids in the homologous sequences VAR2CSA-FCR3

### Statistical analysis

The analyses included singleton deliveries only. Differences in antibody levels between groups were analysed by nonparametric methods (Mann–Whitney or Kruskal–Wallis tests). p values were corrected for multiple comparisons by Holm method using R function p adjust. Adjusted p values < 0.05 were considered significant.

To estimate if antibody levels at enrollment and at gestational week 30–32 predict reduced risk of pregnancy loss and PTD, the Lunn & McNeil Competing Risks Model (LMCRM) [24] were fitted. Three distinct modelling approaches were utilized for antibody levels as predictor: (A) the mean log_10_ antibody levels, (B) all 11 antibodies in 1 model, and (C) each antibody in 11 different models. A likelihood ratio test confirmed that approach (A) was more parsimonious than approach (B) without information loss and approach (C) gave similar results to approach (A), and results of model (A) are presented. Because of the longitudinal nature of the study, gestational age was used as the time variable. All models were adjusted for the same covariates. Time dependent variables were used for covariates cumulative IPTp and malaria infection during follow up visits, and malaria at the time of antibody measurement. In addition, the model was adjusted for gravidity and insecticide treated net (ITN) use. The analysis was conducted in R with packages survival and data table.

Multivariate logistic regression models were used for each cross-sectional analysis (enrollment, gestational week 30–32 and delivery) to examine the relationships between antibody levels to VAR2CSA and LBW or SGA. Normal birthweight or normal weight for gestational age served as the reference group. Multivariate linear regression models were used for each cross-sectional analysis (enrollment, gestational week 30–32 and delivery) to examine the relationships between antibody levels to VAR2CSA and birthweight or gestational age. Models were adjusted for gravidity, malaria infection, ITN use and number of IPTp doses. Models fitted for the analysis of birthweight and LBW were also adjusted for gestational age.

Multinomial logistic regression was used to examine the relationships between antibody levels and maternal anaemia at enrollment, and multivariate linear regression model was used to examine the relationships between antibody levels and hemoglobin. The 3 levels of the categorical outcome were defined as no anaemia, mild anaemia (haemoglobin levels 10–10.9 g/dl), and moderate/severe anaemia (haemoglobin levels < 10 g/dl). No anaemia served as the reference group. The predictors include mean log_10_ antibody levels with adjustment for malaria infection at enrollment, insecticide-treated bed net (ITN) use, and gestational age.

## Results

### Study population

The study population included women at enrollment (n = 657), at gestational week 30–32 (n = 515), and at delivery (n = 656) (Table 2). Antibody levels were measured on 603, 437 and 590 samples collected at enrollment, at gestational week 30–32 and at delivery respectively. Fewer women were included in the antibody analysis during gestational week 30–32 for the following reasons: some women suffered miscarriage, stillbirth or preterm delivery prior to gestational week 30–32; women who enrolled around 30–32 weeks were categorized as enrollment; some samples had not been shipped to the US by the time of assay. Gestational age at recruitment was similar between women of different gravidities, regardless of their infection status. At enrollment, malaria infection prevalence detected by blood smear was significantly higher in primigravid and secundigravid than multigravid women (p < 0.0001 and p = 0.01 respectively), and higher in primigravid than secundigravid women (p = 0.03). At delivery, infection prevalence was higher in primigravidae than multigravidae (p = 0.003); infection rate at delivery was similar between secundigravidae and multigravidae. The lower prevalence of infection at delivery compared to enrollment could be due to IPTp-SP, and to treatment of malaria infections during longitudinal follow up. 67.1, 73.7 and 76.2% of primigravidae, secundigravidae and multigravidae, respectively, received 2 or more doses of IPTp-SP, and 55, 43 and 33% of primigravidae, secundigravidae and multigravidae, respectively, received at least one anti-malarial treatment for an infection. Overall, 57.9, 47.6 and 34.2% of primigravidae, secundigravidae and multigravidae respectively had at least one infection detected by blood smear microscopy during the study. Primigravidae experienced between 0 and 5 (average 0.8) infections, secundigravidae experienced between 0 and 4 (average 0.6) infections and multigravidae had between 0 and 3 (average 0.4) infections. Among blood smear negative (BS−) women, the proportions with submicroscopic infections were similar between gravidity groups at enrollment (19.8, 23.5 and 25.4% in primigravidae, secundigravidae and multigravidae respectively), and at delivery (10.4, 13.2 and 16.4% in primigravidae, secundigravidae and multigravidae respectively).

**Table 2:** Study population stratified by gravidity and malaria infection status

	Group	PM status	n (%)	Gestational age (weeks)^a^ Mean (SD)	*p* value^b^
Enrollment	Primigravid	PM−	104	21.4 (7.1)	0.8
		PM+	67 (39.2%)	22.1 (4.7)	
	Secundigravid	PM−	92	20.3 (6.7)	0.2
		PM+	34 (27.0%)	22.1 (5.5)	
	Multigravid	PM−	302	21.1 (6.3)	0.7
		PM+	58 (16.1%)	21.6 (6.6)	
Gestational week 30–32	Primigravid	PM−	122		
		PM+	14 (10.3%)		
	Secundigravid	PM−	90		
		PM+	4 (4.3%)		
	Multigravid	PM−	270		
		PM+	15 (5.3%)		
Delivery	Primigravid	PM−	144	37.6 (4.2)	0.03
		PM+	27 (15.8%)	36.6 (4.1)	
	Secundigravid	PM−	109	37.7 (5.0)	0.8
		PM+	16 (12.8%)	38.6 (1.3)	
	Multigravid	PM−	326	38.6 (4.0)	0.2
		PM+	34 (9.4%)	39.6 (1.3)	
Pregnancy outcome^c^	Primigravid	Pregnancy loss	18 (61.1% BS +)		
		PTD	26 (65.4% BS +)		
		LBW	33 (66.6% BS +)		
		SGA	18 (44.4% BS +)		
	Secundigravid	Pregnancy loss	9 (33.3% BS +)		
		PTD	14 (42.9% BS +)		
		LBW	19 (52.6% BS +)		
		SGA	11 (63.6% BS +)		
	Multigravid	Pregnancy loss	17 (23.5% BS +)		
		PTD	8 (37.5% BS +)		
		LBW	20 (20.0% BS +)		
		SGA	22 (28.6% BS +)		

Pregnancy loss includes miscarriages, stillbirths and early neonatal death

*PTD* Preterm delivery, *LBW* low birthweight, *SGA* small for gestational age

^a^Gestational age at delivery includes viable singleton births

^b^Comparison of gestational age between PM− and PM+ within gravidity group by Mann–Whitney test

^c^Percentage of women that were infected during pregnancy

The proportions of preterm deliveries were higher in primigravid and secundigravid than multigravid women (p < 0.0001 and p = 0.0001 respectively), and the proportion of pregnancy losses was higher in primigravid than multigravid women (p = 0.007).

### Antibody levels differ between gravidity groups

**Fig. 1:**
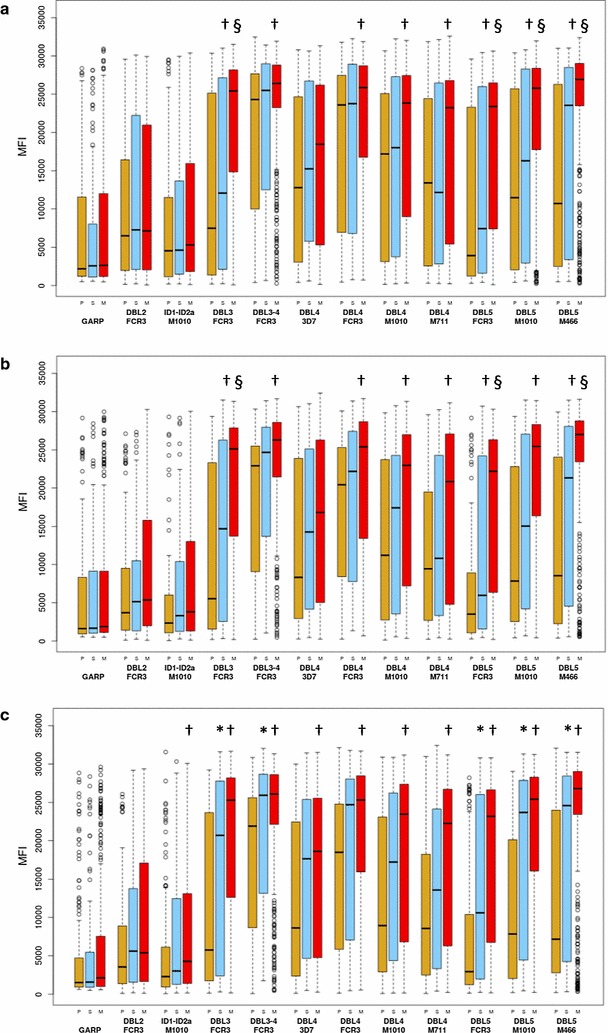
Antibody levels to VAR2CSA domains stratified by gravidity. Levels at enrollment (**a**), gestational week 30–32 (**b**), and delivery (**c**) among primigravid (P, brown boxes), secundigravid (S, blue boxes) and multigravid (M, red boxes) women. Numbers that follow indicate the number of samples at enrollment, gestational week 30–32 and delivery in each gravid group: primigravidae (n = 157, 109, 152); secundigravidae (n = 118, 83, 114); multigravidae (n = 328, 245, 324). Significant differences between primigravidae and multigravidae are indicated with †, between primigravidae and secundigravidae with *, and between secundigravidae and multigravidae are indicated with §. The results are expressed as median fluorescent intensity (MFI). Box plot indicates the median (horizontal line) and interquartile range (box), the whiskers indicate the 10th and 90th percentiles

Gravidity-dependent differences in antibody levels to VAR2CSA domains were observed at the different timepoints (Fig. 1). At enrollment and at gestational week 30–32, antibody levels were significantly higher in multigravidae than primigravidae to most of the domains except DBL2, ID1-ID2a and DBL4-3D7. At delivery, antibody levels to all the domains except DBL2 were higher in multigravidae compared to primigravidae. Multigravidae had higher antibody levels to DBL3 and DBL5 domains than secundigravidae at enrollment and gestational week 30–32. By delivery however, secundigravidae had similar antibody levels as multigravidae, and had higher levels than primigravidae to the same domains that were differentially reactive between secundigravidae and multigravidae at enrollment (Fig. 1). Antibody levels to glutamic acid rich protein (GARP), a protein that is not unique to placental parasites, were similar at the three timepoints between women regardless of gravity (Fig. 1).

Seroprevalence to most VAR2CSA domains was above 70% except for ID1-ID2a and DBL4-3D7 domains in all women and did not significantly change during pregnancy (Table 3). Unlike gravidity-dependent differences in antibody levels, seropositivity was significantly higher in multigravidae compared to primigravidae and secundigravidae to only four domains and one domain, respectively (Table 3).

**Table 3:** Antibodies seropositivity at enrollment, gestational week 30–32 and delivery

	Domain	Primigravid (%)	Secundigravid (%)	Multigravid (%)
Enrollment		n = 157	n = 118	n = 328
	DBL2 (FCR3 allele)	81.5	83.1	84.8
	ID1-DBL2-ID2a (M1010 allele)	61.1	64.4	67.7
	DBL3 (FCR3 allele)	73.9^a^	85.6	93.9
	DBL3-4 (FCR3 allele)	75.8^a^	77.1	89.9
	DBL4 (3D7 allele)	42.0	48.3	53.4
	DBL4 (FCR3 allele)	74.5	71.2^a^	86.9
	DBL4 (M1010 allele)	91.7	91.5	97.6
	DBL4 (M711 allele)	71.3	72.0	82.9
	DBL5 (FCR3 allele)	75.8^a^	82.2	92.7
	DBL5 (M1010 allele)	87.3^a^	94.1	97.6
	DBL5 (M466 allele)	89.8	96.6	96.3
	GARP	77.7	72.9	76.5
Gestational week 30-32		n = 109	n = 83	n = 245
	DBL2 (FCR3 allele)	77.1	75.9	83.7
	ID1-DBL2-ID2a (M1010 allele)	47.7	55.4	62.0
	DBL3 (FCR3 allele)	76.1^a^	79.5	93.5
	DBL3-4 (FCR3 allele)	74.3	80.7	86.5
	DBL4 (3D7 allele)	34.9	47.0	51.0
	DBL4 (FCR3 allele)	76.1	73.5	81.2
	DBL4 (M1010 allele)	90.8	95.2	96.7
	DBL4 (M711 allele)	70.6	74.7	79.6
	DBL5 (FCR3 allele)	73.4^a^	80.7	91.8
	DBL5 (M1010 allele)	87.2	92.8	95.9
	DBL5 (M466 allele)	93.6	94.0	96.3
	GARP	64.2	68.7	74.3
Delivery		n = 152	n = 114	n = 324
	DBL2 (FCR3 allele)	75.7	81.6	81.8
	ID1-DBL2-ID2a (M1010 allele)	48.7	60.5	59.9
	DBL3 (FCR3 allele)	77.6^a^	81.6	93.5
	DBL3-4 (FCR3 allele)	72.4^a^	80.7	87.7
	DBL4 (3D7 allele)	35.5^a^	50.9	54.3
	DBL4 (FCR3 allele)	72.4	71.9	83.6
	DBL4 (M1010 allele)	90.8	91.2	95.4
	DBL4 (M711 allele)	68.4	73.7	80.6
	DBL5 (FCR3 allele)	76.3^a^	78.9	90.7
	DBL5 (M1010 allele)	88.2	90.4	94.8
	DBL5 (M466 allele)	90.8	93.9	96.0
	GARP	64.5	64.0	69.4

^a^Seropositivity significantly lower compared to multigravidae, adjusted p value < 0.05

### Infection increases antibody levels in a gravidity-dependent pattern

**Fig. 2:**
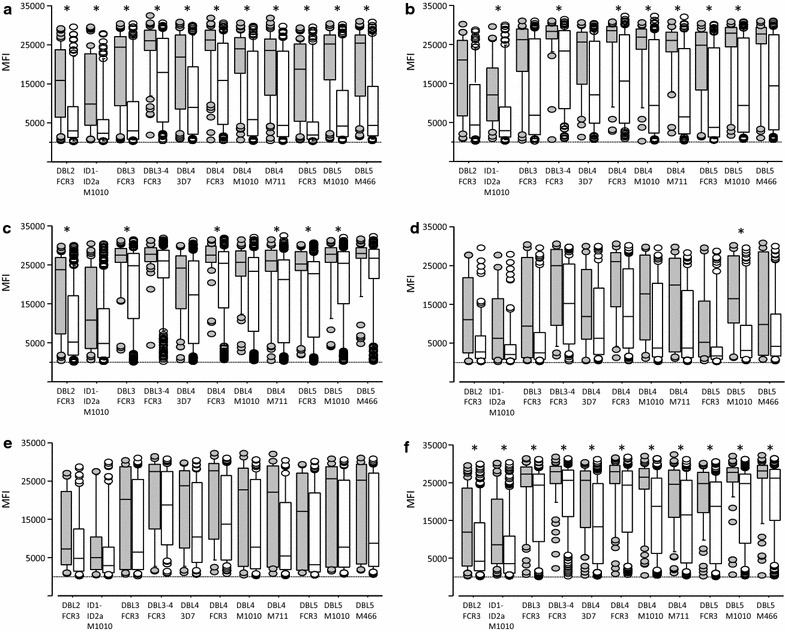
Antibody levels to VAR2CSA domains in malaria infected (grey boxes) and uninfected women (open boxes) at enrollment in primigravid (**a**, **d**), secundigravid (**b**, **e**) and multigravid (**c**, **f**) women. Infections detected by blood smear microscopy are shown in **a**–**c**, and submicroscopic infections detected by PCR in **d**–**f**. Numbers that follow indicate the number of samples with positive blood smear or positive by PCR and with no infection in each gravid group: primigravidae (**a** n = 63, 94; **d** n = 18, 73); secundigravidae (**b** n = 29, 89; **e** n = 19, 62); multigravidae (**c** n = 50, 278; **f** n = 65, 190). Significant differences in antibody levels in malaria-infected women versus uninfected indicated by an asterisk. The results are expressed as median fluorescent intensity (MFI). Box plot indicates the median (horizontal line) and interquartile range (box), the whiskers indicate the 10th and 90th percentiles

Malaria infections are generally known to increase antibody levels. Here, we related VAR2CSA antibody levels to acute infections detected by blood smear microscopy at different times during pregnancy, and to submicroscopic infections detected by nested PCR (peripheral blood at enrollment and gestational week 30–32 and at delivery by placental blood or peripheral blood when placental samples were not available). Among primigravidae, antibody levels at enrollment were significantly higher to all VAR2CSA domains in BS+ versus BS− women (Fig. 2). In secundigravidae and multigravidae, infection detected by blood smear microscopy at enrollment increased antibody levels against 7 and 6 of the 11 domains, respectively (Fig. 2). Antibody levels were similar between malaria-infected and uninfected primigravidae, secundigravidae or multigravidae at gestational week 30–32 and at delivery (Additional file 1).

The effect of past infections on antibody levels at gestational week 30–32 and at delivery was further evaluated. Women with BS− at gestational week 30–32 and at delivery were stratified into two groups: women infected at least once prior to antibody measurement defined as “past infection”, and women without any positive blood smear prior to antibody measurement defined as “no past infection” (Additional file 2). At gestational week 30–32, antibody levels to the 11 VAR2CSA domains were significantly higher in primigravidae with past infection versus no past infection. At delivery, primigravidae with past infection had significantly higher antibody levels to domains DBL2, DBL3-4, DBL4-FCR3, DBL4-M1010, DBL4-M711, DBL5-FCR3 and DBL5-M1010. In secundigravidae, antibody levels to domains DBL3-4, DBL4-FCR3, DBL4-M1010, DBL4-M711, DBL5-FCR3, DBL5-M1010 and DBL4-M466 were significantly higher among women with a past infection and multigravidae with a past infection had a significantly higher antibody levels to DBL2 domain (Additional file 2).

Median antibody levels to VAR2CSA domains were uniformly higher in women with submicroscopic infections versus uninfected women, stratified by parity (Fig. 2d–f). However, while these differences were significant for all domains in analyses of multigravidae, in primigravidae the differences were only significant for the maternal isolate M1010 allele of DBL5, and none were significant for secundigravidae.

As described above, antibody levels to VAR2CSA domains increase with gravidity and are higher in infected women at enrollment (but not at later timepoints); further analysis examined whether infection modifies gravidity-dependent differences in antibody levels. Among women with negative blood smears at enrollment, at gestational week 30–32 and at delivery, differences between primigravidae and multigravidae were similar to the differences observed in the comparison between primigravidae and multigravidae in the non-stratified analysis (Fig. 1). Among women with positive blood smears (BS+) at enrollment, multigravidae had significantly higher antibody levels (Holm adjusted p < 0.05) than primigravidae for 1/1 DBL3 domain (FCR3 allele) and 2/3 DBL5 domains (FCR3 and maternal isolate M466), but not of the other 7 domains. Further, antibody levels did not differ between primigravidae and multigravidae who were BS+ at gestational week 30–32 or at delivery, and there were no differences between secundigravidae and multigravidae who were BS+ at any of the time-points.

Secundigravidae had DBL3 and DBL5 reactivities similar to primigravidae at enrollment and at gestational week 30–32, and reactivities similar to multigravidae at delivery. This pattern could be attributed to malaria experience during the second pregnancy. To test this hypothesis, women who were blood smear-negative (BS−) at delivery were stratified based on whether they had at least one infection detected by blood smear microscopy prior to delivery. Among women with no infections during pregnancy, reactivities in secundigravidae to DBL3 and DBL5 domains were similar to those in primigravidae but lower than those in multigravidae. Conversely, among women with at least one infection during pregnancy, reactivities in secundigravidae to DBL3 and DBL5 domains were now higher than those in primigravidae and similar to those in multigravidae (Additional file 2).

Antibody levels at enrollment and gestational week 30–32 did not predict a reduction in infection at delivery. This was true both for primigravidae (OR (95% CI) 0.94 (0.30–3.09), and 1.73 (0.24–15.75), respectively, for antibody levels at enrollment and at gestational week 30–32), and for multigravidae (OR (95% CI) 0.79 (0.35–1.93) and 0.51 (0.22–1.25).

### Changes in IgG levels during pregnancy

**Fig. 3:**
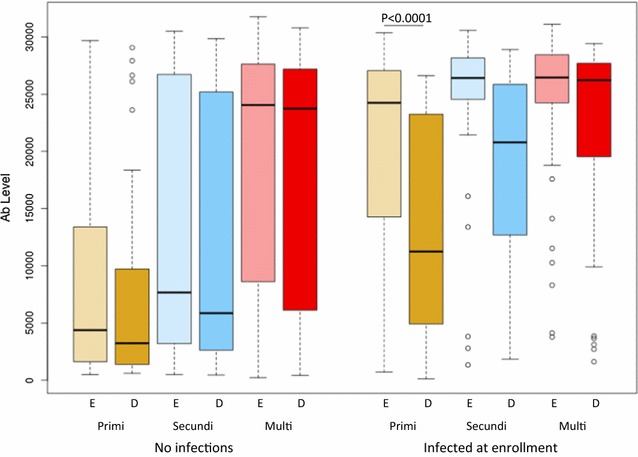
Antibody levels to VAR2CSA domains expressed as mean antibody levels to 11 domains at enrollment (E, light shade) and at delivery (D, dark shade) in women with no infections during the study and women infected at enrollment only. Numbers that follow indicate the number of samples with no infection or infection at enrollment only at enrollment (E) and at delivery (D) in each gravid group: primigravidae (E, n = 66, 48; D, n = 64, 46); secundigravidae (E, n = 64, 22; D, n = 57, 23); multigravidae (E, n = 212, 38; D, n = 204, 40). Box plot indicates the median (horizontal line) and interquartile range (box), the whiskers indicate the 10th and 90th percentiles

Previous studies reported that antibody reactivities to the IE surface of CSA-binding parasites in primigravidae and to recombinant VAR2CSA in both primigravidae and multigravidae declined after pregnancy [25, 26]. In this cohort, infection at enrollment was associated with higher antibody levels to all VAR2CSA recombinants (in primigravidae) or some VAR2CSA recombinants (in secundigravidae and multigravidae), raising the possibility that these antibody titers would decline in the absence of additional infection. To answer this question, antibody levels at enrollment and at delivery were compared in women who were BS+ versus BS− at enrollment, and had no further infections detected throughout pregnancy. Among BS+ primigravidae, antibody levels to 10/11 VAR2CSA domains but not to the control protein GARP, declined sharply and were significantly lower at delivery, p < 0.05 (Fig. 3 and Additional file 2), suggesting that malaria infection in primigravidae elicits short-lived IgG. Of note, BS+ and BS− primigravidae included in this analysis had similar duration of followup during pregnancy (mean 14.4 and 15.9 weeks, respectively, p = 0.5). Among secundigravidae and multigravidae, antibody levels were similar between enrollment and delivery (p > 0.05), suggesting long-lived IgG (Fig. 3 and Additional file 2).

### Antibodies to VAR2CSA domains do not predict a reduction in preterm delivery and pregnancy loss

Antibody levels measured at enrollment and at gestational week 30–32 were related to preterm delivery and pregnancy loss. Because antibody levels to the different domains were highly correlated (Additional file 3), the average (log_10_) of the antibody levels to the 11 VAR2CSA domains was used as a composite predictor. Average VAR2CSA antibody level was assessed as a predictor for a reduction in poor pregnancy outcomes after adjustment for gravidity, malaria infection, IPTp, and insecticide-treated net usage. In this analysis, the mean antibody levels to VAR2CSA domains was not associated with reduced risk of preterm delivery, HR (95% CI) 1.08 (0.51–2.30), p = 0.8 or reduced risk of pregnancy loss, HR (95% CI) 1.13 (0.55–2.34), p = 0.7. To ensure that average antibody levels to the various domains did not mask associations with any individual domains, antibody levels to each domain as predictors were separately analysed. Similar to the results seen with the model of average antibody levels, antibody levels to individual domains did not predict reduced risk of pregnancy loss or preterm delivery.

Potential limitation of this analysis is the number of pregnancy loss and preterm delivery cases (6.8 and 7.8% of the pregnancies respectively). Because of the similar effect of antibody levels on pregnancy loss and preterm delivery (p = 0.97), and both outcomes reflect early pregnancy termination, further analysis for the combined outcomes (n = 90) was performed. Mean antibody levels to VAR2CSA domains were not associated with reduced risk of combined preterm delivery and pregnancy loss, HR (95% CI) 1.1 (0.64–1.91), p = 0.7.

### Antibodies to VAR2CSA domains do not predict increases in birthweight or gestational age

The association between antibody levels measured at enrollment, at gestational week 30–32, and at delivery, with birth outcomes was further examined. Mean antibody levels to the 11 VAR2CSA domains at enrollment and gestational week 30–32 did not predict a reduction in LBW or SGA, and antibody levels at delivery were not associated with these outcomes (Table 4).

**Table 4:** Regression analysis of mean antibody levels to 11 VAR2CSA domains during pregnancy and at delivery on birth weight and gestational age

Sampling time	Outcome	Gravidity^a^	OR (95% CI)	p value
Enrollment	LBW	Primigravid	1.84 (0.43–9.20)	0.4
		Multigravid	0.84 (0.30–2.53)	0.7
	SGA	Primigravid	1.97 (0.62–7.093)	0.3
		Multigravid	0.58 (0.25–1.44)	0.2
Gestational week 30–32	LBW	Primigravid	7.06 (0.61–131.45)	0.1
		Multigravid	0.72 (0.23–2.45)	0.6
	SGA	Primigravid	1.36 (0.24–8.50)	0.7
		Multigravid	0.69 (0.27–1.99)	0.5
Delivery	LBW	Primigravid	3.34 (0.70–19.47)	0.2
		Multigravid	0.66 (0.25–1.87)	0.4
	SGA	Primigravid	2.34 (0.59–11.11)	0.3
		Multigravid	0.76 (0.34–1.88)	0.5

Sampling time	Outcome	Gravidity	β	p value
Enrollment	BW	Primigravid	− 0.14	0.02
		Multigravid	0.02	0.7
	GA	Primigravid	− 0.77	0.07
		Multigravid	− 0.05	0.8
Gestational week 30–32	BW	Primigravid	− 0.16	0.07
		Multigravid	− 0.01	0.8
	GA	Primigravid	− 0.84	0.1
		Multigravid	0.24	0.2
Delivery	BW	Primigravid	− 0.13	0.03
		Multigravid	− 0.02	0.7
	GA	Primigravid	− 0.53	0.2
		Multigravid	− 0.02	0.9

Models were adjusted for malaria infection, number of IPTp doses and ITN. The analyses of LBW and BW were also adjusted for gestational age

*LBW* low birthweight, *SGA* small for gestational age, *BW* birthweight, *GA* gestational age

^a^Primigravid, 1st pregnancy; Multigravid, ≥ 1 previous pregnancies

In primigravidae but not in multigravidae, antibody levels at enrollment, at gestational week 30–32 and at delivery were negatively associated with birthweight (Table 4). Similarly, in primigravidae, antibody levels were negatively associated with gestational age, but these relationships did not achieve statistical significance (Table 4).

### Antibodies to VAR2CSA domains do not predict a reduction in maternal anaemia

In this longitudinal study, pregnant women received iron supplementation after enrolling into the study. Therefore, the analysis focused on the association between antibody levels measured at enrollment with maternal anaemia at enrollment. Because few women had severe anaemia, moderate and severe anaemia were analysed as one group defined as hemoglobin less than 10 g/dl. Mild anaemia was defined as haemoglobin level of 10–10.9 g/dl [27]. Increased antibody levels were associated with reduced haemoglobin levels and increased odds of having moderate/severe anaemia in both primigravidae and multigravidae, but these associations did not achieve statistical significance (Table 5).

**Table 5:** Regression analysis of mean antibody levels to VAR2CSA domains at enrollment on maternal anemia

Outcome	Gravidity^a^	Adjusted OR (95% CI)^b^	p value
Mild anemia	Primigravid	0.88 (0.29–2.71)	0.8
	Multigravid	1.93 (1.04–3.58)	0.04
Moderate/severe anemia	Primigravid	1.92 (0.69–5.36)	0.2
	Multigravid	1.69 (0.92–3.12)	0.09

Outcome	Gravidity	Adjusted β^b^	p value
Hemoglobin	Primigravid	− 0.05	0.9
	Multigravid	− 0.19	0.2

^a^Primigravid, 1st pregnancy; Multigravid, ≥ 1 previous pregnancies

^b^Models were adjusted for malaria infection, ITN and gestational age

## Discussion

Over successive pregnancies, women develop antibodies against IE surface proteins expressed by placental parasites that block parasite adhesion to the placental receptor CSA [7]. The presence of anti-adhesion antibodies has been associated with increased birth weight and gestational age [12, 28]. Anti-adhesion antibodies broadly react with placental IE regardless of their geographical origin, indicating that they target antigenically conserved epitopes [7]. IE adhesion to CSA is mediated by VAR2CSA, the leading candidate for developing a vaccine to prevent PM. In the current study, the effects of gravidity and infection on VAR2CSA antibody levels during pregnancy, and the associations between VAR2CSA antibody levels and pregnancy outcomes were examined.

Similar to previous reports, antibody levels to VAR2CSA domains DBL2, DBL3, DBL4 and DBL5 but not to the control protein GARP increased with gravidity [11, 12]. Antibody levels between VAR2CSA domains correlated, but not between antibody levels to VAR2CSA and the control protein GARP (Additional file 3). Patent infection at enrollment was associated with an increase in VAR2CSA antibody levels (Fig. 2), a difference that was not observed at gestational week 30–32 and at delivery. However, among women who were BS− at week 30–32 or at delivery, those who had been BS+ at any earlier timepoint often had higher antibody levels, but this was primarily seen in the paucigravid groups: for primigravidae, antibody levels were higher to 11/11 and 8/11 domains at the two respective timepoints; for secundigravidae, 0/11 and 8/11; for multigravidae, 0/11 and 1/11 (Additional file 2). These results are consistent with a previous study in Mozambique reporting that at delivery, antibody levels to domains DBL2, DBL3 and DBL5 were higher in women with histological evidence of acute or past infection than women without evidence of infection [11].

Submicroscopic infections at enrollment were associated with a significant increase in antibody levels to all VAR2CSA domains in multigravidae, but to only one domain in primigravidae. In mice, subpatent infections with *Plasmodium chabaudi chabaudi* induce antibody responses to conserved antigens, but not to variant surface antigen, and this has been ascribed to several possible causes including relatively low variants surface antigen dose [29]. Here, submicroscopic infections probably represent a low antigen dose sufficient to boost existing immune responses to the variant antigen in multigravidae but not to elicit new antibodies in primigravidae.

An earlier study conducted in northwestern Thailand, a low malaria transmission area described that antibody levels to malaria antigens including VAR2CSA declined during pregnancy in both infected and uninfected women. However, the half-life of antibodies to VAR2CSA was much longer than the half-life of antibodies to merozoite antigens (36–157 years) [30]. Another study of non-pregnant women residing in an area with stable malaria transmission, described a progressive increase during pregnancy in VAR2CSA-specific IgG levels and B cell numbers in both primigravidae and multigravidae, and then a decline postpartum [26]. Despite the rapid decline in antibody levels post-delivery, gravidity-dependent differences in antibody levels to VAR2CSA were observed in non-pregnant women suggesting an increased longevity of plasma cells secreting IgG over successive pregnancies [31]. Consistent with this observation, in the current study, antibody levels rapidly declined and were significantly lower at delivery in primigravidae who were infected at enrollment but not thereafter, indicating that short-lived antibody is induced in primigravidae, who are relatively naïve.

At enrollment and at gestational week 30–32, secundigravidae had antibody levels that were similar to those of primigravidae, but were lower than those of multigravidae to four domains. By delivery, secundigravidae who experienced at least one infection had similar antibody levels as multigravidae. These results suggest that at least one infection during the 2nd pregnancy is required to achieve antibody levels similar to those of clinically resistant multigravidae.

Two vaccine candidates based on the N-terminal region of VAR2CSA are currently being evaluated in clinical trials. One product, PRIMALVAC, is based on the DBL1-DBL2 domain combination of 3D7 allele, and the other, PAMVAC, is based on the ID1-ID2a region of FCR3 allele [32]. These fragments were selected for placental malaria vaccine (PMV) development based on their binding affinity to CSA [33, 34]. In addition, these fragments elicited heterologous functional antibodies that inhibited IE binding to CSA [35, 36]. Conflicting results have been reported on naturally acquired antibodies to the N-terminal VAR2CSA fragments. In Cameroon, antibody levels to ID1-ID2a were similar between men and pregnant women [37]. In Benin and in the current study, antibody levels to DBL1-DBL2 were significantly higher in multigravidae than primigravidae [12]. Variation in allelic forms, domain boundaries, timing of antibody measurement and assay among others complicates direct comparisons between studies, that may be addressed by harmonizing these variables.

Antibody levels to VAR2CSA domains were not associated with reduced risk of pregnancy loss and preterm delivery, nor with increased gestational age and birthweight or decreased maternal anaemia. The negative association between birthweight and antibody levels in primigravidae could be explained by the increase in antibody levels during malaria infection. The results here confirm that the recombinant VAR2CSA domains including those containing the minimal CSA-binding domain used in this study are reactive to malaria-induced antibodies, but may fail to re-create the epitopes targeted by antibodies associated with protection. In a recent study, multigravidae plasma depleted of antibodies directed to the domains evaluated here, did not reduce the level of functional anti-adhesion antibodies, but reduced the level of reactivity with IE surface measured by flow cytometry [22]. Induction of functional anti-adhesion antibodies is sensitive to changes in domain boundaries and allelic type [36], which may explain the lack of an association between antibodies to domains evaluated here including those containing the minimal CSA-binding region and pregnancy outcomes.

## Conclusion

In summary, malaria infection increases VAR2CSA antibody levels early in pregnancy, but infected and uninfected women did not differ in antibody levels during third trimester or at delivery. During infection, primigravidae had similar VAR2CSA antibody levels as multigravidae. However, antibody levels after an infection declined sharply in primigravidae while remaining stable in multigravidae, indicating that VAR2CSA antibody responses are short-lived in susceptible primigravidae but durable in resistant multigravidae. In multivariate analyses, VAR2CSA antibody levels were not associated with improved pregnancy outcomes, providing additional evidence that recombinant VAR2CSA domains and fragments may not re-create protective epitopes.

## Additional files

**Additional file 1: Figure S1.** Antibody levels to VAR2CSA domains in malaria infected (grey boxes) and uninfected women (open boxes) at gestational week 30-32 (A), and delivery (B) in primigravid (P), secundigravid (S) and multigravid (M) women.

**Additional file 2: Table S1.** Comparison of antibody levels at gestational week 30-32 (A) and at delivery (B) between women with and without past infection stratified by gravidity. **Table S2.** Comparison of antibody levels at delivery between gravid groups with and without past infection. **Table S3.** Comparison of antibody levels between enrollment and delivery in primigravidae with and without detectable infections at enrollment.

**Additional file 3: Table S4.** Correlations between antibody levels to VAR2CSA domains stratified by gravidity.

## Abbreviations

LBW: low birthweight
SGA: small for gestational age
PTD: preterm delivery
PMV: placental malaria vaccine
IPTp: intermittent preventive treatment in pregnancy
GARP: glutamic acid rich protein
