# Supplementary material for: Antibody levels to recombinant VAR2CSA domains vary with Plasmodium falciparum parasitaemia, gestational age, and gravidity, but do not predict pregnancy outcomes
Source: Malar J. 2018 Mar 9;17:106. doi: 10.1186/s12936-018-2258-9 (PMC5845157; doi:10.1186/s12936-018-2258-9)
Supplement: Supplementary file 1 — Additional file 1: Figure S1. Antibody levels to VAR2CSA domains in malaria infected (grey boxes) and uninfected women (open boxes) at gestational week 30-32 (A), and delivery (B) in primigravid (P), secundigravid (S) and multigravid (M) women. [file 12936_2018_2258_MOESM1_ESM.pptx]

## Slide 1
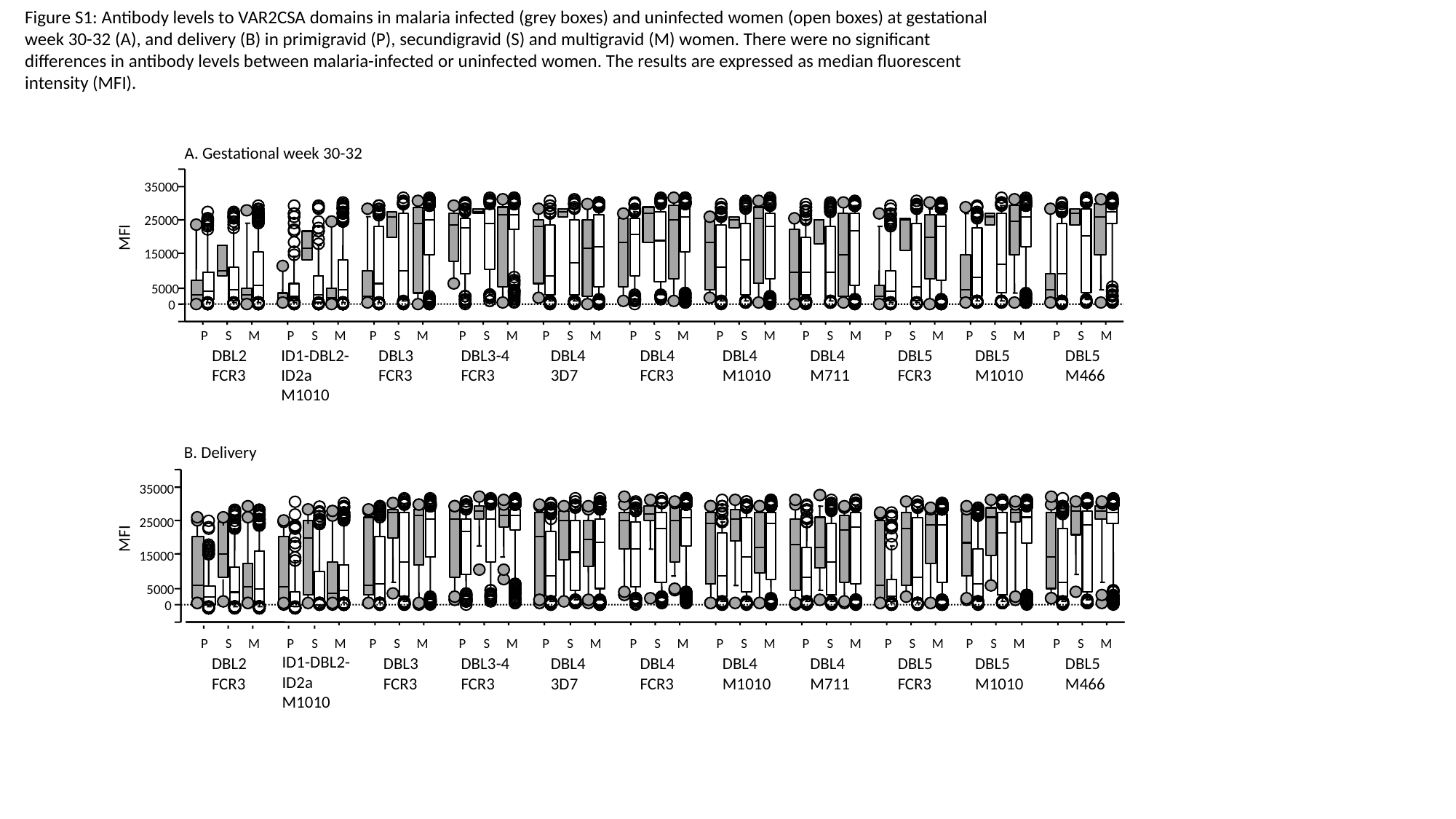

Figure S1: Antibody levels to VAR2CSA domains in malaria infected (grey boxes) and uninfected women (open boxes) at gestational week 30-32 (A), and delivery (B) in primigravid (P), secundigravid (S) and multigravid (M) women. There were no significant differences in antibody levels between malaria-infected or uninfected women. The results are expressed as median fluorescent intensity (MFI).
A. Gestational week 30-32
35000
25000
MFI
15000
5000
0
P
S
M
P
S
M
P
S
M
P
S
M
P
S
M
P
S
M
P
S
M
P
S
M
P
S
M
P
S
M
P
S
M
DBL2
FCR3
ID1-DBL2-
ID2a
M1010
DBL3
FCR3
DBL3-4
FCR3
DBL4
3D7
DBL4
FCR3
DBL4
M1010
DBL4
M711
DBL5
FCR3
DBL5
M1010
DBL5
M466
B. Delivery
35000
25000
MFI
15000
5000
0
P
S
M
P
S
M
P
S
M
P
S
M
P
S
M
P
S
M
P
S
M
P
S
M
P
S
M
P
S
M
P
S
M
ID1-DBL2-
ID2a
M1010
DBL2
FCR3
DBL3
FCR3
DBL3-4
FCR3
DBL4
3D7
DBL4
FCR3
DBL4
M1010
DBL4
M711
DBL5
FCR3
DBL5
M1010
DBL5
M466
