# Supplementary material for: Antibody levels to recombinant VAR2CSA domains vary with Plasmodium falciparum parasitaemia, gestational age, and gravidity, but do not predict pregnancy outcomes
Source: Malar J. 2018 Mar 9;17:106. doi: 10.1186/s12936-018-2258-9 (PMC5845157; doi:10.1186/s12936-018-2258-9)
Supplement: Supplementary file 2 — Additional file 2: Table S1. Comparison of antibody levels at gestational week 30-32 (A) and at delivery (B) between women with and without past infection stratified by gravidity. Table S2. Comparison of antibody levels at delivery between gravid groups with and without past infection. Table S3. Comparison of antibody levels between enrollment and delivery in primigravidae with and without detectable infections at enrollment. [file 12936_2018_2258_MOESM2_ESM.docx]

Table S1. Comparison of antibody levels at gestational week 30-32 (A) and at delivery (B) between women with and without past infection stratified by gravidity.

A

| Domain | No past infection | Had past infection | Adj. P value |
| --- | --- | --- | --- |
|  | Mean (SD) | Mean (SD) |  |
| Primigravid (n=52, 47) | | | |
| DBL2 FCR3 | 4497 (6058) | 9322 (7947) | 0.002 |
| ID1-ID2a | 3309 (5053) | 6766 (7073) | 0.002 |
| DBL3 FCR3 | 6754 (9069) | 15990 (9707) | <0.0001 |
| DBL3-4 FCR3 | 14144 (9477) | 22077 (7167) | 0.0007 |
| DBL4 3D7 | 8383 (8940) | 15356 (10226) | 0.006 |
| DBL4 FCR3 | 12760 (9511) | 21373 (7027) | 0.0005 |
| DBL4 M1010 | 7539 (8334) | 17376 (8857) | <0.0001 |
| DBL4 M711 | 6939 (7553) | 15606 (8737) | <0.0001 |
| DBL5 FCR3 | 4294 (6856) | 9346 (7703) | <0.0001 |
| DBL5 M1010 | 72767 (8459) | 16592 (8999) | <0.0001 |
| DBL5 M466 | 7559 (8486) | 17188 (9692) | <0.0001 |
|  | | | |
| Secundigravid (n=56, 24) | | | |
| DBL2 FCR3 | 7336 (7744) | 8952 (8249) | NS |
| ID1-ID2a M1010 | 6181 (8148) | 6042 (5865) | NS |
| DBL3 FCR3 | 12642 (11325) | 18354 (11531) | NS |
| DBL3-4 FCR3 | 18518 (9576) | 24659 (7424) | NS |
| DBL4 3D7 | 14342 (10423) | 14310 (10427) | NS |
| DBL4 FCR3 | 15953 (10696) | 22521 (7757) | NS |
| DBL4 M1010 | 12849 (10422) | 18968 (9481) | NS |
| DBL4 M711 | 10910 (10146) | 17329 (9403) | NS |
| DBL5 FCR3 | 9163 (10457) | 15430 (10590) | NS |
| DBL5 M1010 | 12770 (10991) | 20703 (10806) | NS |
| DBL5 M466 | 14685 (11475) | 21721 (10706) | NS |
|  | | | |
| Multigravid (n=191, 41) | | | |
| DBL2 FCR3 | 8571 (8608) | 12079 (10162) | NS |
| ID1-ID2a M1010 | 8103 (8723) | 9026 (9609) | NS |
| DBL3 FCR3 | 20518 (9804) | 20746 (9771) | NS |
| DBL3-4 FCR3 | 22837 (8570) | 23947 (6808) | NS |
| DBL4 3D7 | 15705 (10891) | 17244 (10163) | NS |
| DBL4 FCR3 | 20984 (9858) | 22172 (8616) | NS |
| DBL4 M1010 | 17367 (10578) | 18645 (9365) | NS |
| DBL4 M711 | 16744 (10859) | 18330 (10474) | NS |
| DBL5 FCR3 | 17067 (10268) | 18370 (10012) | NS |
| DBL5 M1010 | 21177 (9623) | 22278 (9113) | NS |
| DBL5 M466 | 22712 (9309) | 23369 (8742) | NS |

B

| Domain | No past infection  Mean (SD) | Had past infection  Mean (SD) | Adj. P value |
| --- | --- | --- | --- |
|  |  |  |  |
| Primigravid (n=65, 60) | | | |
| DBL2 FCR3 | 4101 (5025) | 6605 (6571) | 0.046 |
| ID1-ID2a M1010 | 3763 (5208) | 5501 (7341) | NS |
| DBL3 FCR3 | 8377 (9682) | 12881 (10172) | 0.04 |
| DBL3-4 FCR3 | 14953 (9542) | 19883 (8728) | 0.03 |
| DBL4 3D7 | 9206 (8916) | 13373 (9655) | NS |
| DBL4 FCR3 | 13029 (9802) | 17792 (8648) | 0.048 |
| DBL4 M1010 | 8637 (8853) | 14143 (8881) | 0.002 |
| DBL4 M711 | 8595 (8898) | 12043 (8496) | 0.046 |
| DBL5 FCR3 | 5275 (7897) | 7552 (7647) | 0.01 |
| DBL5 M1010 | 8301 (9530) | 12552 (9529) | 0.03 |
| DBL5 M466 | 8626 (9324) | 13762 (10675) | NS |
|  | | | |
| Secundigravid (n=55, 44) | | | |
| DBL2 FCR3 | 7066 (8277) | 10252 (8856) | NS |
| ID1-ID2a M1010 | 6317 (8544) | 9040 (9115) | NS |
| DBL3 FCR3 | 12445 (12057) | 19732 (10893) | NS |
| DBL3-4 FCR3 | 17844 (10694) | 24465 (7030) | 0.046 |
| DBL4 3D7 | 13203 (10083) | 17142 (11120) | 0.048 |
| DBL4 FCR3 | 15434 (11874) | 22749 (8310) | 0.045 |
| DBL4 M1010 | 12152 (10537) | 19291 (10021) | 0.03 |
| DBL4 M711 | 10907 (10625) | 17376 (9704) | 0.046 |
| DBL5 FCR3 | 9945 (11542) | 17467 (10592) | 0.02 |
| DBL5 M1010 | 12609 (12020) | 22155 (9323) | 0.01 |
| DBL5 M466 | 13952 (12075) | 22803 (9393) | 0.03 |
|  | | | |
| Multigravid (n=203, 88) | | | |
| DBL2 FCR3 | 8481 (8954) | 12277 (9768) | 0.03 |
| ID1-ID2a M1010 | 7793 (8687) | 9476 (9755) | NS |
| DBL3 FCR3 | 19727 (10405) | 21846 (9402) | NS |
| DBL3-4 FCR3 | 22020 (8986) | 24433 (6664) | NS |
| DBL4 3D7 | 15128 (10859) | 17339 (9737) | NS |
| DBL4 FCR3 | 20437 (10065) | 23332 (7767) | NS |
| DBL4 M1010 | 17162 (10824) | 19240 (10042) | NS |
| DBL4 M711 | 16045 (11100) | 19012 (9892) | NS |
| DBL5 FCR3 | 16407 (10741) | 19007 (9862) | NS |
| DBL5 M1010 | 20080 (10338) | 22889 (8668) | NS |
| DBL5 M466 | 21510 (10147) | 24339 (8276) | NS |

Table S2. Comparison of antibody levels at delivery between gravid groups with and without past infection

| No past infection | | | | |  | | Had a past infection | | |
| --- | --- | --- | --- | --- | --- | --- | --- | --- | --- |
|  | Primigravid  (n=65) | Multigravid (n=204) | |  | | Primigravid (n=63) | | Multigravid  (n=89) |  |
| Domain | Mean (SD) | Mean (SD) | Adj.  P value |  | | Mean (SD) | | Mean (SD) | Adj. P value |
| DBL2 FCR3 | 4040 (5010) | 8545 (9010) | 0.01 |  | | 6994 (6575) | | 12211 (9660) | NS |
| ID1-ID2a M1010 | 3706 (5187) | 7761 (8727) | 0.009 |  | | 5826 (7452) | | 9548 (9660) | NS |
| DBL3 FCR3 | 8251 (9660) | 19773 (10396) | <0.0001 |  | | 13347 (10071) | | 21922 (9396) | <0.0001 |
| DBL3-4 FCR3 | 14725 (9645) | 22056 (8971) | <0.0001 |  | | 20385 (8259) | | 24488 (6665) | 0.006 |
| DBL4 3D7 | 9066 (8918) | 15197 (10896) | 0.008 |  | | 13709 (9603) | | 17462 (9719) | NS |
| DBL4 FCR3 | 12830 (9856) | 20478 (10052) | <0.0001 |  | | 18423 (8351) | | 23400 (7769) | 0.0009 |
| DBL4 M1010 | 8506 (8847) | 17205 (10791) | <0.0001 |  | | 14297 (8752) | | 19332 (10064) | 0.008 |
| DBL4 M711 | 8464 (8891) | 16103 (11111) | 0.0001 |  | | 12242 (8441) | | 19120 (9889) | 0.0005 |
| DBL5 FCR3 | 5196 (7861) | 16464 (10737) | <0.0001 |  | | 7584 (7476) | | 19105 (9884) | <0.0001 |
| DBL5 M1010 | 8176 (9508) | 20124 (10327) | <0.0001 |  | | 12812 (9278) | | 22951 (8659) | <0.0001 |
| DBL5 M466 | 8496 (9310) | 21547 (10126) | <0.0001 |  | | 14025 (10399) | | 24395 (8275) | <0.0001 |
| GARP | 5048 (7088) | 5856 (7869) | NS |  | | 5447 (7585) | | 6462 (8279) | NS |
|  |  |  |  |  | |  | |  |  |
|  | Primigravid (n=65) | Secundigravid  (n=57) | |  | | Primigravid (n=63) | | Secundigravid  (n=45) | |
| DBL2 FCR3 | 4040 (5010) | 7022 (8147) | NS |  | | 6994 (6575) | | 10142 (8786) | NS |
| ID1-ID2a M1010 | 8496 (9310) | 6186 (8423) | NS |  | | 5826 (7452) | | 8941 (9036) | NS |
| DBL3 FCR3 | 3706 (5187) | 12106 (11976) | NS |  | | 13347 (10071) | | 19933 (10852) | 0.02 |
| DBL3-4 FCR3 | 8251 (9660) | 17775 (10626) | NS |  | | 20385 (8259) | | 24350 (6992) | 0.03 |
| DBL4 3D7 | 14725 (9645) | 13299 (10030) | NS |  | | 13709 (9603) | | 16876 (11137) | NS |
| DBL4 FCR3 | 9066 (8918) | 15279 (11707) | NS |  | | 18423 (8351) | | 22611 (8268) | NS |
| DBL4 M1010 | 12830 (9856) | 12333 (10470) | NS |  | | 14297 (8752) | | 19457 (9969) | NS |
| DBL4 M711 | 8506 (8847) | 10877 (10476) | NS |  | | 12242 (8441) | | 17612 (9723) | NS |
| DBL5 FCR3 | 8464 (8891) | 9714 (11401) | NS |  | | 7584 (7476) | | 17694 (10581) | <0.0001 |
| DBL5 M1010 | 5196 (7861) | 12443 (11877) | NS |  | | 12812 (9278) | | 22308 (9273) | <0.0001 |
| DBL5 M466 | 8176 (9508) | 13657 (11966) | NS |  | | 14025 (10399) | | 22949 (9337) | 0.0004 |
| GARP | 5048 (7088) | 4368 (6141) | NS |  | | 5447 (7585) | | 5789 (7768) | NS |
|  |  |  |  |  | |  | |  |  |
|  | Secundigravid (n=57) | Multigravid  (n=204) | |  | | Secundigravid (n=45) | | Multigravid (n=89) |  |
| DBL2 FCR3 | 7022 (8147) | 8545 (9010) | NS |  | | 10142 (8786) | | 12211 (9660) | NS |
| ID1-ID2a M1010 | 6186 (8423) | 7761 (8727) | NS |  | | 8941 (9036) | | 9548 (9660) | NS |
| DBL3 FCR3 | 12106 (11976) | 19773 (10396) | 0.005 |  | | 19933 (10852) | | 21922 (9396) | NS |
| DBL3-4 FCR3 | 17775 (10626) | 22056 (8971) | NS |  | | 24350 (6992) | | 24488 (6665) | NS |
| DBL4 3D7 | 13299 (10030) | 15197 (10896) | NS |  | | 16876 (11137) | | 17462 (9719) | NS |
| DBL4 FCR3 | 15279 (11707) | 20478 (10052) | NS |  | | 22611 (8268) | | 23400 (7769) | NS |
| DBL4 M1010 | 12333 (10470) | 17205 (10791) | NS |  | | 19457 (9969) | | 19332 (10064) | NS |
| DBL4 M711 | 10877 (10476) | 16103 (11111) | NS |  | | 17612 (9723) | | 19120 (9889) | NS |
| DBL5 FCR3 | 9714 (11401) | 16464 (10737) | 0.005 |  | | 17694 (10581) | | 19105 (9884) | NS |
| DBL5 M1010 | 12443 (11877) | 20124 (10327) | 0.02 |  | | 22308 (9273) | | 22951 (8659) | NS |
| DBL5 M466 | 13657 (11966) | 21547 (10126) | 0.004 |  | | 22949 (9337) | | 24395 (8275) | NS |
| GARP | 4368 (6141) | 5856 (7869) | NS |  | | 5789 (7768) | | 6462 (8279) | NS |

Table S3. Comparison of antibody levels between enrollment and delivery in primigravidae with and without detectable infections at enrollment

|  | No infections^1^ | | | Infected at enrollment^2^ | | |
| --- | --- | --- | --- | --- | --- | --- |
| Domain | Enrollment  Mean (SD) | Delivery  Mean (SD) | P value | Enrollment  Mean (SD) | Delivery  Mean (SD) | P value |
|  | n=66 | n=64 |  | n=48 | n=46 |  |
| DBL2 FCR3 | 6834 (8246) | 4101 (5025) | NS | 15558 (8628) | 6475 (6316) | <0.0001 |
| ID1-ID2a M1010 | 6208 (7910) | 3763 (5208) | NS | 13356 (9570) | 5082 (6354) | <0.0001 |
| DBL3 FCR3 | 9236 (10608) | 8377 (9682) | NS | 20723 (9439) | 15048 (10014) | NS |
| DBL3-4 FCR3 | 16480 (10509) | 14953 (9542) | NS | 24669 (5620) | 19432 (8395) | 0.003 |
| DBL4 3D7 | 11500 (10129) | 9206 (8916) | NS | 18491 (9364) | 12266 (9365) | 0.02 |
| DBL4 FCR3 | 15201 (10831) | 13029 (9802) | NS | 24960 (5875) | 18240 (8442) | 0.0006 |
| DBL4 M1010 | 10948 (10760) | 8637 (8853) | NS | 21764 (7639) | 13169 (8217) | <0.0001 |
| DBL4 M711 | 11255 (10423) | 8595 (8898) | NS | 20173 (8320) | 12122 (8534) | 0.0009 |
| DBL5 FCR3 | 6689 (9348) | 5275 (7897) | NS | 17195 (9943) | 7983 (7316) | 0.002 |
| DBL5 M1010 | 9915 (10418) | 8301 (9530) | NS | 22234 (8808) | 13614 (9139) | 0.0004 |
| DBL5 M466 | 10279 (10234) | 8626 (9324) | NS | 21616 (9181) | 14990 (10218) | 0.02 |
| GARP | 6925 (8675) | 5110 (7761) | NS | 8029 (9400) | 5514 (7761) | NS |

^1^ No microscopically detected infection

^2^ Microscopically detected infections only at enrollment
